# A randomised controlled trial of succinylated gelatin (4%) fluid on urinary acute kidney injury biomarkers in cardiac surgical patients

**DOI:** 10.1186/s40635-021-00412-9

**Published:** 2021-09-22

**Authors:** Lisa Smart, Corrin Boyd, Edward Litton, Warren Pavey, Philip Vlaskovsky, Umar Ali, Trevor Mori, Anne Barden, Kwok Ming Ho

**Affiliations:** 1 College of Science, Health, Engineering and Education, Murdoch University, South St, Murdoch, WA 6150 Australia; 2 Centre for Clinical Research in Emergency Medicine, Perth, Australia; 3 Intensive Care Unit, Fiona Stanley Hospital, Murdoch, Australia; 4 Intensive Care Unit, St John of God Hospital, Subiaco, Australia; 5 Department of Anaesthesia, Fiona Stanley Hospital, Murdoch, Australia; 6 Medical School, University of Western Australia, Perth, Australia; 7 Department of Cardiothoracic Surgery and Transplantation, Fiona Stanley Hospital, Murdoch, Australia; 8 Intensive Care Unit, Royal Perth Hospital, Perth, Australia

**Keywords:** Cardiac surgery, Colloid, Fluid resuscitation, Renal, Urine

## Abstract

**Background:**

Fluid resuscitation is frequently required for cardiac surgical patients admitted to the intensive care unit. The ideal fluid of choice in regard to efficacy and safety remains uncertain. Compared with crystalloid fluid, colloid fluid may result in less positive fluid balance. However, some synthetic colloids are associated with increased risk of acute kidney injury (AKI). This study compared the effects of succinylated gelatin (4%) (GEL) with compound sodium lactate (CSL) on urinary AKI biomarkers in patients after cardiac surgery.

**Methods:**

Cardiac surgical patients who required an intravenous fluid bolus of at least 500 mL postoperatively were randomly allocated to receive GEL or CSL as the resuscitation fluid of choice for the subsequent 24 h. Primary outcomes were serial urinary neutrophil gelatinase-associated lipocalin (NGAL) and cystatin C concentrations measured at baseline, 1 h, 5 h and 24 h after enrolment, with higher concentrations indicating greater kidney injury. Secondary biomarker outcomes included urinary clusterin, α1-microglobulin and F_2_-isoprostanes concentrations. Differences in change of biomarker concentration between the two groups over time were compared with mixed-effects regression models. Statistical significance was set at *P* < 0.05.

**Results:**

Forty cardiac surgical patients (*n* = 20 per group) with similar baseline characteristics were included. There was no significant difference in the median volume of fluid boluses administered over 24 h between the GEL (1250 mL, Q1–Q3 500–1750) and CSL group (1000 mL, Q1–Q3 500–1375) (*P* = 0.42). There was a significantly greater increase in urinary cystatin C (*P* < 0.001), clusterin (*P* < 0.001), α1-microglobulin (*P* < 0.001) and F_2_-isoprostanes (*P* = 0.020) concentrations over time in the GEL group, compared to the CSL group. Change in urinary NGAL concentration (*P* = 0.68) over time was not significantly different between the groups. The results were not modified by adjustment for either urinary osmolality or EuroSCORE II predicted risk of mortality.

**Conclusions:**

This preliminary randomised controlled trial showed that use of succinylated gelatin (4%) for fluid resuscitation after cardiac surgery was associated with increased biomarker concentrations of renal tubular injury and dysfunction, compared to crystalloid fluid. These results generate concern that use of intravenous gelatin fluid may contribute to clinically relevant postoperative AKI.

*Trial registration* ANZCTR.org.au, ACTRN12617001461381. Registered on 16th October, 2017, http://www.anzctr.org.au/Trial/Registration/TrialReview.aspx?id=373619&isReview=true.

**Supplementary Information:**

The online version contains supplementary material available at 10.1186/s40635-021-00412-9.

## Background

Optimising cardiac preload with intravenous fluid boluses is frequently required in cardiac surgical patients admitted to the intensive care unit (ICU). However, positive fluid balance and fluid overload are associated with adverse patient outcomes [1, 2], and the ideal fluid remains uncertain. Compared with crystalloids, there is some evidence that synthetic colloids provide both greater volume expansion and cardiac output, and a less positive fluid balance [3–5], and that these perceived benefits influence reported practice [6]. Conversely, the safety of some types of colloid fluid such as hydroxyethyl starch remains a concern, including their potential to induce or exacerbate acute kidney injury (AKI) [7]. Diversion of clinical practice away from hydroxyethyl starch may have increased use of gelatin colloids in some cardiac surgical centres [8], despite a lack of efficacy and safety data on gelatin colloids from large randomised controlled trials.

Patients undergoing cardiac surgery are at high risk of postoperative AKI [9], a major cause of increased morbidity and mortality. As such, using an intravenous fluid that is safe for the kidney is pivotal for these patients. Recent observational data suggest that gelatin fluid may induce or exacerbate renal injury [10, 11]. Animal models have shown that gelatin fluid causes renal tubular epithelial vacuolation, or vesiculation, and an increase in biomarkers of AKI including neutrophil gelatinase-associated lipocalin (NGAL) and cystatin C [12–14]. These biomarkers are associated with clinically significant AKI in patients after cardiac surgery [15–17]. Furthermore, gelatin fluid induces platelet dysfunction and reduces clot strength [18–20], which is particularly important for patients undergoing cardiac surgery. Given these potential adverse effects, it is currently unclear if administration of gelatin fluid results in overall benefit or harm.

In this randomised controlled trial, we compared the effects of succinylated gelatin (4%) (GEL) (Gelofusine™, B. Braun Australia Pty Ltd) on a range of urinary AKI biomarkers, compared to a balanced crystalloid solution, compound sodium lactate (CSL), in patients who had undergone major cardiac surgery. We hypothesised that fluid bolus therapy with CSL would be associated with a lesser increase in concentrations of urinary NGAL and cystatin C over time, compared to GEL. Secondary outcomes included additional urinary biomarkers of AKI; clusterin, α1-microglobulin and F_2_-isoprostanes concentrations.

## Methods

### Trial design and participants

The GELATIne Fluid and Acute Kidney Injury in Critical Illness (GELATI) Trial was an investigator-initiated, single-centre, randomised, open-label, parallel-arm designed clinical trial. Approval was obtained from the South Metropolitan Health Service Human Research Ethics Committee (RGS0000000105; 31st August, 2017), and registration with the Australian and New Zealand Clinical Trials Registry (ACTRN12617001461381; 16th October, 2017) before commencement. After obtaining written informed consent prior to surgery, 40 participants (20 in each group) were enrolled and randomly allocated to receive either GEL or CSL for fluid boluses after cardiac surgery in the 30-bed ICU of the Fiona Stanley Hospital in Perth, Western Australia.

After postoperative admission to the ICU, consented patients were screened again by a study investigator, in conjunction with the ICU clinician. Eligible patients were those for whom the treating ICU clinician intended to administer an intravenous fluid bolus of 500 mL or more within 60 min, and in whom they believed there was equipoise in using either GEL or CSL. The exclusion criteria were: < 18 years old; serum creatinine > 265 µmol L^−1^ prior to surgery; end-stage renal failure requiring dialysis; urine output < 10 mL h^−1^ over the previous 4 h; severe AKI requiring renal replacement therapy (RRT), or likely to require RRT within the next 24 h; known allergy to GEL; received GEL in the previous 48 h; admitted to ICU for longer than 96 h; previously enrolled in the study; a limitation of care order had been placed; death was perceived to be imminent; or the treating clinician perceived that participation in the study was not in the patient’s best interests. The serum creatinine concentration limit of 265 µmol L^−1^, or 3 mg dL^−1^, corresponded with an estimated creatinine clearance of < 30 mL/min for the anticipated demographic, consistent with severe chronic renal insufficiency [21].

Intraoperative management was conducted according to local standard practice. All participants received routine monitoring including 5-lead electrocardiogram, systemic arterial and central venous pressure monitoring. Pulmonary artery catheterisation was performed at the treating anaesthetist’s discretion. For participants receiving cardiopulmonary bypass (CPB), 2 g of tranexamic acid was administered as an intravenous infusion, commencing after induction, and heparin was dosed to achieve an activated clotting time (ACT) of > 480 s. CPB circuits were primed with 1600 mL of balanced isotonic crystalloid fluid (Plasmalyte A; Baxter Healthcare, Phoenixville, PA, USA). Following CPB, heparin was reversed with protamine targeting an ACT of < 130 s. For participants not receiving CPB, administration of drugs was similar except that target ACT was > 300 s during heparinisation and < 150 s post-reversal. Fluid management in the operating theatre consisted of blood products and balanced isotonic crystalloid, as required, but no synthetic colloid fluid.

### Randomisation and blinding

Patients were randomly allocated to receive intravenous fluid bolus therapy with either GEL or CSL. Randomisation was performed via block design of 4 blocks of 10 participants each (5 in each treatment arm) generated by an online random number generator 1. Allocation concealment was achieved by sealed, opaque, sequential numbered envelopes prepared by a scientist not involved with the study. Once a participant was enrolled, either a member of clinical staff or study investigator opened the next available envelope to reveal treatment allocation. The administration of study fluid was open label for reasons of feasibility, but primary outcome assessment was blinded to clinical staff.

### Interventions

Participants were randomised to receive an initial study fluid bolus of at least 500 mL. Fluid delivery started as soon as a baseline urine sample had been collected. Further boluses of the assigned study fluid were administered if considered clinically indicated by the treating clinician. After administering a total study fluid volume of 50 mL kg^−1^ or 3000 mL, or 24 h after randomisation, whichever occurred first, the choice of further fluid therapy was at the discretion of the treating clinician. The use of more than 50 mL kg^−1^ or 3000 mL of GEL was strongly discouraged. Use of other fluids during the intraoperative and postoperative period, including crystalloid for maintenance fluid requirements or drug administration, and blood products including human albumin solution, was at the discretion of the treating clinicians.

### Outcomes and analytic techniques

The primary outcome was urinary NGAL and cystatin C concentrations over time up to 24 h post-randomisation. Secondary outcomes included other biomarkers of AKI: urinary clusterin, α1-microglobulin and F_2_-isoprostanes concentrations. Urine collection occurred immediately after randomisation (T0), then at 1 (T1), 5 (T5) and 24 (T24) hours after the start of the first fluid bolus. Details of the sample collection methods are provided in Additional file 1. Urinary NGAL, cystatin C, clusterin and α1-microglobulin were measured using a commercial multiplex magnetic bead array (Milliplex Human Renal Toxicity Panel, MilliporeSigma, Burlington, MA, USA) according to manufacturer’s instructions. Urine F_2_-isoprostanes concentration, which indicates induced oxidative stress, was measured by gas chromatography–mass spectrometry (Agilent 6890N gas chromatograph coupled to an Agilent 5975B mass spectrometer) using electron capture negative ionisation and selective ion monitoring. Urine osmolality was measured by freezing point depression (Osmometer Type 6 M, Löser Messtechnik, Berlin, Germany). Urine creatinine concentration was measured using a commercial analyser (COBAS INTEGRA 400 plus, Roche Diagnostics, Basel, Switzerland).

The following clinical outcomes were also recorded in this study: maximum AKI stage within the first 7 days, RRT-free days within the first 28 days, number of blood product units transfused during the ICU stay, ICU-free days within 28 days, and 60-day all-cause mortality. Clinical data collection details are described in Additional file 1.

### Statistical methods

Sample size calculation was based on the clinically important difference in NGAL that has been reported to be associated with AKI in a critically ill population. Patients with burn injury and early AKI had an approximately twofold increase in urine NGAL concentration within 48 h after admission, compared to those without AKI [22]. Extrapolating the means and standard deviations from the data provided in this study (AKI: 240 ± 76 ng/mL; no AKI: 140 ± 38 ng/mL), a sample size of 5 in each arm would give 80% power (*α* = 0.05) to detect a twofold difference in NGAL. A conservative sample size of 20 per arm (total 40 patients) was planned to reduce the risk of type II error, which would detect a mean difference in NGAL > 55 ng.mL^−1^ between the two groups with a power of 80%.

Normality of data was assessed by visual inspection of histograms and Q–Q plots. Data are presented in median and first to third quartiles (Q1–Q3) or counts and percentages, as appropriate. Inferential tests were not applied to baseline characteristics, in line with the 2010 CONSORT statement [23]. Total study fluid volumes were compared using Wilcoxon rank sum test. Biomarker concentrations were log-transformed to produce normal or approximately normal distributions. Comparisons of change in biomarker concentration over time were made using linear mixed-effects models. Models were bootstrapped to obtain robust *P* values for biomarkers that remained slightly skewed following log transformation. P values are presented for the interaction between treatment and time in the unadjusted model. Biomarker concentrations were summarised as predicted mean (95% confidence interval) estimated by the unadjusted models. A Bonferroni-corrected *P* value significance cut-off of < 0.01 for each linear mixed-effects model was further applied in order to reduce Type I error created by multiple biomarker analyses. The effects of urine concentration, as osmolality, and illness severity, as estimated by EuroSCORE II mortality prediction [24], were also assessed as potential confounders.

Clinical outcomes were compared using Fisher’s exact test for categorical variables or Wilcoxon rank sum test for non-normal continuous data. Ventilation time was compared using negative binomial regression due to the nature of its distribution. No adjustment was applied to account for multiple comparisons in the exploratory analyses of clinical outcomes.

Analyses were performed using Stata 14 (College Station, TX, USA) and SAS (SAS Institute, Cary, NC, USA) with significance set at two-sided *P* < 0.05, except for Bonferroni correction where stated.

## Results

### Participant characteristics

**Fig. 1:**
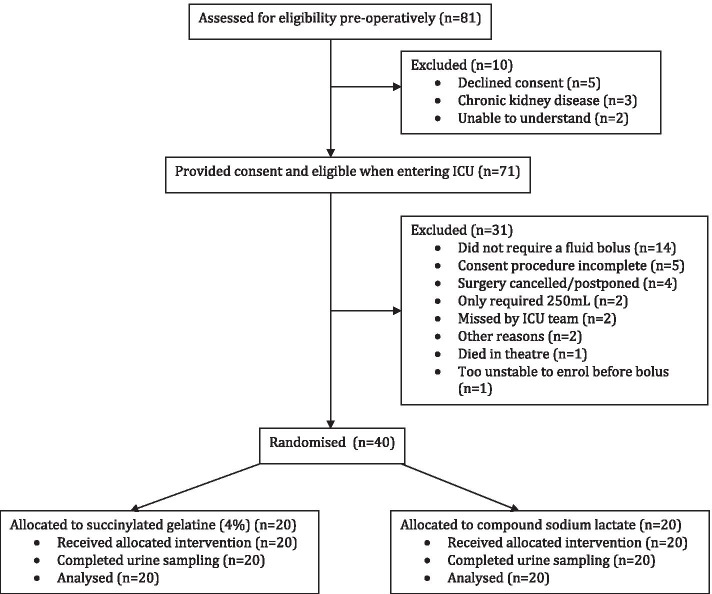
Study CONSORT flowchart

Of the 81 participants screened for eligibility between December, 2018 and February, 2019, 40 participants (*n* = 20 per study arm) were enrolled and randomised (Fig. 1). Baseline characteristics were similar between the two groups (Table 1). The total volume of study fluid administered during the study period was not significantly different between the two groups (*P* = 0.42) (Table 2). No participants received more than a total of 2500 mL of study fluid. Three participants (two in the GEL group and one in the CSL group) received a single bolus of crystalloid in the ICU before study enrolment. One participant in the GEL group returned to the operating theatre and received a crystalloid bolus during reoperation between T1 and T5. No participants in the CSL group received GEL during their entire ICU stay. Thirty-eight out of 40 participants had their urine samples collected per protocol; in one participant, the T1 urine sample was delayed by 1 h and, in another participant, the T24 sample was delayed by four hours.

**Table 1:** Characteristics of cardiac surgical patients who were randomised to receive either succinylated gelatin (4%) (GEL) or compound sodium lactate (CSL) as resuscitation fluid after cardiac surgery

Characteristic	GEL (*n* = 20)	CSL (*n* = 20)
Age (years)	67 (59–70)	68 (58–72)
Male, *n* (%)	14 (70)	17 (85)
Body weight (kg)	82 (72–97)	81 (70–96)
NYHA class, *n* (%)		
1	12 (60)	13 (65)
2	3 (15)	5 (25)
3	5 (25)	2 (10)
LVEF (%)	56 (46–63)	60 (45–63)
Preoperative serum creatinine concentration (μmol/L)	76 (70–93)	82 (65–98)
Procedure type, *n* (%)		
CABG	11 (55)	14 (70)
Valve replacement	5 (25)	4 (20)
Other	4 (20)	2 (10)
EuroSCORE II mortality predicted risk (%)	1.24 (0.79–1.82)	1.03 (0.82–1.95)
CPB, *n* (%)	15 (75)	14 (70)
Pump time (mins)*	103 (82–144)	98 (76–110)
Cross clamp time (mins)*	86 (47–122)	55 (47–75)
Time-to-enrol** (mins)	75 (36–266)	114 (41–391)
Urine output at enrolment (mL/kg/h)	0.6 (0.3–1.7)	0.8 (0.3–1.5)

Data are presented as either median (Q1–Q3) or number (percentage). *Sample size differs to heading of columns. ** Time from ICU admission to randomisation

CABG, coronary artery bypass graft; CPB, cardiopulmonary bypass; NYHA, New York Heart Association; LVEF, left ventricular ejection fraction

**Table 2:** Median (Q1–Q3) volumes of study fluid (mL) delivered to cardiac surgical patients who were randomised to receive succinylated gelatin (4%) (GEL) or compound sodium lactate (CSL) as fluid resuscitation therapy after cardiac surgery between different urine sampling time points

Outcome	T0–T1	T1–T5	T5–T24	Total	*P* value^a^
Fluid volume (mL)					
GEL	500 (500–500)	125 (0–500)	250 (0–1000)	1250 (500–1750)	
CSL	500 (500–500)	125 (0–500)	250 (0–500)	1000 (500–1375)	0.42

^a^Difference between treatment groups in total volume of fluid given during study period (Wilcoxon rank-sum test)

### Serial changes in AKI biomarkers

**Fig. 2:**
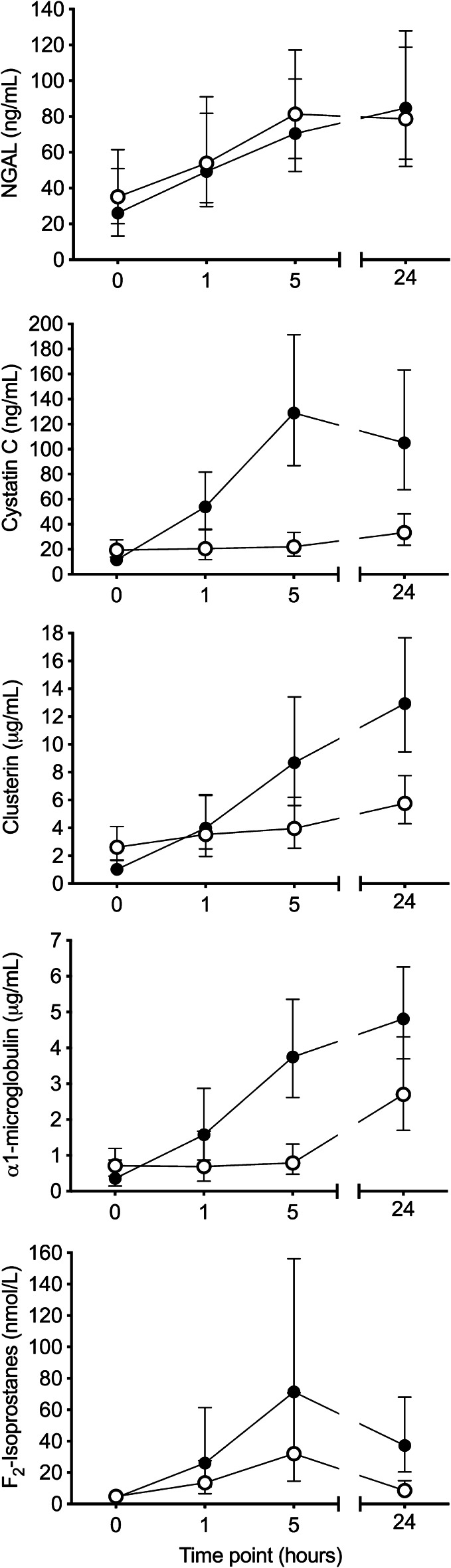
Urinary biomarker concentrations (predicted mean, 95% confidence interval) neutrophil gelatinase-associated lipocalin (NGAL) (**a**), cystatin C (**b**), clusterin (**c**), α1-microglobulin (**d**) and F_2_-isoprostanes (**e**) of cardiac surgical patients who were randomised to receive either succinylated gelatine (4%) (closed circles) or compound sodium lactate (open circles) as fluid resuscitation therapy after cardiac surgery. Urine was sampled before the intervention (0 h), then 1, 5 and 24 h later

Of the five urinary biomarkers analysed, there was a significantly greater increase over time for cystatin C (*P* < 0.001), clusterin (*P* < 0.001), α1-microglobulin (*P* < 0.001) and F_2_-isoprostanes (*P* = 0.020) concentrations in the GEL group, compared to the CSL group (Fig. 2). The differences between groups for cystatin C, clusterin and α1-microglobulin remained significant after adjusting for multiple comparisons. There was no significant difference in change of NGAL concentration over time between groups (*P* = 0.68). The overall result for each biomarker was not modified by adjustment for urine osmolality and there was no association detected between biomarker concentrations and EuroSCORE II predicted risks of mortality. Results of the linear mixed-effects model analysis for the primary outcomes, both unadjusted and adjusted for osmolality, are provided in Additional file 2. Urine concentration and output are also described in Figure, Additional file 3.

### Clinical outcomes

According to rises in plasma creatinine during the study period, 13 participants developed clinical AKI during the first 7 days of hospitalisation (five in the GEL group, eight in the CSL group) (*P* = 0.73) (Table 3). Biomarker concentrations stratified by clinical AKI were explored post hoc and are provided in Additional file 4. No patients required RRT within 28 days. The median fluid balance between T0 and T24 was not significantly different between the two groups (GEL: 1751 mL (872–3022) vs CSL: 1261 mL (688–2214)) (*P* = 0.27). However, there were more participants in the GEL group that received packed red blood cell transfusions, compared to the CSL group (5 in the GEL group, none in the CSL group) (*P* = 0.047). The other clinical outcomes were not significantly different between the two groups (Table 3).

**Table 3:** Clinical outcomes of cardiac surgical patients who were randomised to receive succinylated gelatin (4%) (GEL) or compound sodium lactate (CSL) as fluid resuscitation therapy after cardiac surgery

Outcome	GEL (*n* = 20)	CSL (*n* = 20)	*P* value**
KDIGO stage*, *n* (%)			0.73
Stage I	4 (20)	7 (35)	–
Stage II	1 (5)	1 (5)	–
Stage III	0	0	–
28-day RRT-free days	28 (28–28)	28 (28–28)	N/A
Chest drain output (mL)	485 (344–687)	335 (268–428)	0.05
Received blood products, *n* (%)			
Packed red blood cells	5 (25)	0 (0)	0.05
Cryoprecipitate	1 (5)	0 (0)	N/A
Platelet concentrate	1 (5)	0 (0)	N/A
Received albumin, *n* (%)	2 (10)	2 (10)	N/A
Return to theatre, *n* (%)	3 (15)	0 (0)	0.23
Ventilation time (hours)	17 (11–30)	11 (10–15)	0.06
Fluid balance (mL)	1751 (872–3022)	1261 (688–2214)	0.27
28-day ICU-free-days	26 (24–27)	26 (24–27)	0.60
60-day mortality, *n* (%)	0 (0)	0 (0)	N/A

Data are presented as either median (Q1–Q3) or number of patients (percentage of group). * KDIGO stage was calculated as maximum change in plasma creatinine within 7 days of randomisation. **P values have been rounded to two decimal places

ICU, intensive care unit; KDIGO, Kidney Disease Outcomes Kidney Disease: Improving Global Outcomes; RRT, renal replacement therapy

## Discussion

This randomised controlled trial showed that using intravenous gelatin for fluid resuscitation, instead of crystalloid fluid, after cardiac surgery was associated with a significantly greater increase in urinary biomarkers of AKI over time. These results are concerning as to the safety of gelatin fluid, with potential adverse clinical implications for its use, and require further discussion.

There is a strong biological rationale that urinary AKI biomarkers may be more sensitive for detecting AKI than a rise in plasma creatinine, which defines clinically detectable AKI. Cystatin C and α1-microglobulin are low molecular weight proteins that are freely filtered and reabsorbed in the proximal tubule. Therefore, their elevation in urine is expected in the early stages of proximal tubular dysfunction [25–27]. Both of these biomarkers measured postoperatively are associated with development of clinical AKI in cardiac surgical patients [17, 28]. Clusterin expression is upregulated in both proximal and distal tubules after cellular injury [29, 30], therefore it is also expected to increase in the urine, even with mild AKI. Finally, F_2_-isoprostanes are a marker of cellular oxidative injury and an elevation in urine concentration suggests renal oxidative stress [31]. As such, increases in urinary concentrations of these four biomarkers in patients who had received gelatin are concerning and may represent clinically relevant kidney injury.

We did not observe any differences in urinary NGAL concentration between the two groups. NGAL is a protein rapidly produced by both the proximal and distal renal tubular epithelial cells in response to ischaemia and drug-induced injury [26, 32]. Over a 30-fold increase in urinary NGAL was observed after gelatin in a canine haemorrhagic shock model [14] and this effect was also observed in a rodent sepsis model [12]. It is possible that there is a species difference in either susceptibility to gelatin-induced renal injury or stimulation of NGAL production by gelatin. For the latter mechanism, gelatin is rapidly degraded by gelatinase conjugated to the heterodimeric form of NGAL [33, 34], but it is unclear if provision of the substrate itself (i.e. gelatin) can stimulate production of NGAL in the kidney. Although we did not observe a large increase in urinary NGAL after GEL in this study, compared to CSL, there was a rise in NGAL within hours in both groups over time (Additional file 2), which is consistent with other cardiac surgical studies [28, 35–38]. It has been shown that all forms of NGAL are present postoperatively in cardiac surgical patients [39], therefore any effect of GEL on a certain specific form of NGAL may have been obscured by increases in other types of NGAL. In addition, the presence of gelatin itself in the urine may have interfered with the NGAL assay [40], which may have masked elevations of this biomarker in either group.

Clinical AKI as defined by a substantial rise in plasma creatinine concentration occurring in approximately one-third of participants, with the majority of events being stage 1. This proportion of participants experiencing AKI is consistent with previous studies reporting on similar cohorts [15–17]. Though this event was transient for most, even small-to-moderate increases in creatinine may have adverse consequences for cardiac surgical patients [41], including increased resource utilisation 2. We were interested in associations between increased biomarker concentrations and the presence of clinical AKI in this study, which were explored post hoc (Additional file 4); however, an inadequate sample size of patients with clinical AKI precluded any definitive inference. For patients who do not experience a postoperative rise in plasma creatinine, there may still be adverse renal sequelae. Evidence shows that a proportion of patients undergoing cardiac surgery will have sub-clinical AKI, with elevated urinary biomarkers paired with a lack of rise in plasma creatinine concentration, and only manifested with a subsequent loss of renal functional reserve [42]. Despite being unable to establish the clinical importance of the rise in biomarker concentrations seen in this study, the potential exacerbation of AKI by use of gelatin fluid presents a major public health issue, especially given the burden of AKI on morbidity and mortality of cardiac surgical patients. This study provides rationale that further investigation assessing the safety and effectiveness of intravenous gelatin fluid is required.

We noted that participants who received gelatin were more likely to require packed red blood cell transfusion, compared to those who had received crystalloid fluid alone. This may well represent a chance finding, but gelatin’s deleterious effects on clot formation could also explain this, in part [18–20]. This finding also supports the results of a recent observational trial in cardiac surgical patients, whereby administration of gelatin was associated with increased chest drain output, bleeding events and red blood cell transfusion, compared to crystalloid fluid alone [43]. The effect of gelatin on coagulation and bleeding remains contentious [44], and this deserves further investigation.

This study has some limitations. Lack of blinding of the study fluid to treating clinicians may have affected treatment decisions, though we did not detect any differences in the volume of study fluid administered between groups and no clinicians withdrew their patients from the study after randomisation. We did not measure urinary albumin concentrations in our participants. Albuminuria secondary to glomerular damage may have caused other functional markers to increase, such as cystatin C, due to competition for tubular uptake [45]. Urine samples were allowed to dwell at room temperature in the urinary collection bag for 1 to 4 h before collection. However, the dwell time was unlikely to confound the results of this trial given the sampling protocol fidelity and the chemical stability of these biomarkers under various storage conditions [46–48]. Urinary biomarkers were not indexed to total urine volume, therefore absolute biomarker concentrations may have been affected by tubular flow rate. It was not considered feasible to align measurement of urine volume, as needed for patient care, with study time points. Adjusted analysis and further data pertaining to urine concentration and volume, available in the Additional files, did not suggest that this was important in explaining our results. Finally, subtle baseline imbalances in participant characteristics in this randomised trial may have introduced some degree of confounding to our results. Nonetheless, our a priori statistical analysis plan was robust by comparing the serial changes in biomarker concentrations while adjusting for any differences in baseline concentrations. Furthermore, our results remained unchanged after adjustment for severity of illness, as estimated by the predicted risk of mortality.

In conclusion, this randomised controlled trial showed that use of succinylated gelatin (4%) for fluid resuscitation after cardiac surgery was associated with an increase in some biomarkers of renal tubular injury and dysfunction within 24 h, compared to crystalloid fluid. These results support the concern that use of gelatin fluid may be associated with increasing the risk of post-operative AKI. There was a signal, albeit still limited, that succinylated gelatin (4%) may affect bleeding tendency. Given that succinylated gelatin (4%) is more expensive than crystalloid fluid, an adequately powered randomised controlled trial focused on clinical outcomes is needed to confirm its safety in cardiac surgical patients.

## Supplementary Information

**Additional file 1.** Sample and data collection of cardiac surgical patients who were randomised to receive either succinylated gelatin (4%) or compound sodium lactate as resuscitation fluid therapy after cardiac surgery.

**Additional file 2.** Linear mixed-effects model analysis of the associations between serial change in urinary neutrophil gelatinase-associated lipocalin and cystatin C concentrations and treatment.

**Additional file 3**. Urine data (median, Q1-Q3) from cardiac surgical patients who were randomised to receive succinylated gelatin (4%) (GEL) or compound sodium lactate (CSL) as resuscitation fluid therapy after cardiac surgery. Urine was sampled before the intervention (T0), then 1 h (T1), 5 h (T5) and 24 h (T24) later. There were missing data for urine output at T0 (GEL: *n* = 3; CSL: *n* = 1), T1 (GEL: *n* = 2; CSL: *n* = 1) and T24 (GEL: *n* = 2; CSL: *n* = 3).

**Additional file 4.** Urinary biomarker concentrations (median, Q1-Q3) NGAL, cystatin C, clusterin, α1-microglobulin and F_2_-isoprostanes of cardiac surgical patients who developed acute kidney injury (AKI) (closed squares) or did not develop AKI (open squares). AKI was defined as a maximum KDIGO score > 0 within 7 days of randomisation. Urine was sampled before the intervention (0 h), then 1, 5 and 24 h later. Abbreviations: AKI; acute kidney disease; KDIGO, Kidney Disease Outcomes Kidney Disease: Improving Global Outcomes; NGAL, neutrophil gelatinase-associated lipocalin.

## Data Availability

Data are available upon reasonable request, with mutual agreement as to the purpose of its use.

## Footnotes

1 www.randomizer.org; accessed on the 5th November, 2018.

2 ANZSCTS Cardiac Surgery Database Program National Annual Report 2018.
