# Supplementary material for: A randomised controlled trial of succinylated gelatin (4%) fluid on urinary acute kidney injury biomarkers in cardiac surgical patients
Source: Intensive Care Med Exp. 2021 Sep 22;9:48. doi: 10.1186/s40635-021-00412-9 (PMC8455786; doi:10.1186/s40635-021-00412-9)
Supplement: Supplementary file 1 — Additional file 1. Sample and data collection of cardiac surgical patients who were randomised to receive either succinylated gelatin (4%) or compound sodium lactate as resuscitation fluid therapy after cardiac surgery. [file 40635_2021_412_MOESM1_ESM.docx]

**Supplemental Digital Content 1**

Sample and data collection of cardiac surgical patients who were randomised to receive either succinylated gelatine (4%) or compound sodium lactate as resuscitation fluid therapy after cardiac surgery.

**Urine sample collection**

At the time of randomisation, 5mL of urine was collected from the urinary collection bag before any study fluid administration (T0). The urine collection bag was then emptied before the study fluid was started. At 1 (T1), 5 (T5) and 24 (T24) hours after the start of the first study fluid bolus, a further 5mL of urine was collected from the urinary collection bag. At T1 and T5, the urine collection bag was emptied after the 5mL urine sample was collected. For T5 and T24, collection 1 hour either side was accepted. After T5, nursing staff were advised to empty the urine collection bag and discard the urine as needed; however, 2 hours before T24 staff were asked to refrain from emptying the bag until the research sample was collected. If the urinary catheter had been removed by T24, then voided urine was sampled at T24. All urine samples were placed on ice, then centrifuged at 350G for 5 minutes within 30 minutes of collection. Supernatant was then aliquoted and immediately frozen at -80^o^C for later batched analysis.

**Clinical data collection**

A standardised case report form was used to collect clinical data. Demographic information, volumes of fluid administered, chest drain output, urine output, fluid balance, blood transfusion units, daily plasma creatinine concentration, and ICU hospitalisation time were retrieved from the intensive care unit (ICU) medical record database (MetaVision Suite, iMDsoft). Clinical AKI was defined by a maximum rise in creatinine within 7 days of randomisation in line with Kidney Disease: Improving Global Outcomes (KDIGO) definitions^a^ whereby stage I was defined as 1.5 to 1.9 times baseline or ≥26.5 μmol/l increase, stage II as 2.0 to 2.9 times baseline and stage III as 3.0 times baseline or increase in serum creatinine to ≥353.6 μmol/l, or initiation of renal replacement therapy. Baseline creatinine was taken from measurement made on the day of surgery before, or at, anaesthetic induction. Maximum creatinine concentration was then recorded daily for 7 days from randomisation and the peak creatinine used for KDIGO staging. Urine output was averaged over 2 hours for T0 if the participant had been present in the ICU for more than 2 hours. If the participant was enrolled within an hour of arrival from theatre, then urine output was omitted. If the participant had arrived from theatre between 1 to 2 hours ago, then urine output was taken from the first hour. Urine output was averaged over the previous 4 hours for T5 and over the previous 2 hours for T24. If the urinary catheter had been removed by T24, then the last urine output recorded was used, as long as it was within 4 hours of T24. Chest drain output was summated from hourly outputs recorded between T0 and T24. If the chest drain had been removed by T24, the total volume from T0 was recorded, regardless of time of removal. Fluid balance was calculated from T0 to T24. For participants that were transferred to wards before T24, the last hourly fluid balance was taken from the ICU record at transfer. Total hospitalisation time, renal replacement therapy and mortality data were obtained from the ICU administrative database. Details required for EuroSCORE II^b^ calculation were obtained from the local Australian and New Zealand Society of Cardiac and Thoracic Surgeons Database Program database.

^a^ Kellum JA, Lameire N (2013) Diagnosis, evaluation, and management of acute kidney injury: a KDIGO summary (Part 1). Crit Care 17:204.

^b^ Nashef SA, Roques F, Sharples LD, et al (2012) EuroSCORE II. Eur J Cardiothoracic Surg 41:734-744.
