# Supplementary material for: A randomised controlled trial of succinylated gelatin (4%) fluid on urinary acute kidney injury biomarkers in cardiac surgical patients
Source: Intensive Care Med Exp. 2021 Sep 22;9:48. doi: 10.1186/s40635-021-00412-9 (PMC8455786; doi:10.1186/s40635-021-00412-9)
Supplement: Supplementary file 2 — Additional file 2. Linear mixed-effects model analysis of the associations between serial change in urinary neutrophil gelatinase-associated lipocalin and cystatin C concentrations and treatment. [file 40635_2021_412_MOESM2_ESM.docx]

**Supplemental Digital Content 2**

Linear mixed model analysis of the association between change in urinary neutrophil gelatinase-associated lipocalin and cystatin C concentrations over time and treatment. Cardiac surgical patients were randomised to receive succinylated gelatin (4%) (GEL) or compound sodium lactate (CSL) (n=20 per group) as bolus therapy in the intensive care unit. Urine was sampled at enrolment, and 1 hour, 5 hours and 24 hours later. Multi-variable model analyses are also provided with adjustment for urine concentration (osmolality).

**Neutrophil Gelatinase-Associated Lipocalin**

Number of observations = 60

Number of subjects = 40

Observations per group = 4

| Predictor | β (95% confidence interval) | P value |
| --- | --- | --- |
| Treatment |  |  |
| CSL | Reference group |  |
| GEL | -0.300 (-0.973 – 0.770) | 0.49 |
| Time point |  |  |
| Baseline | Reference group |  |
| 1 hour | 0.429 (-0.100 – 0.872) | 0.050 |
| 5 hours | 0.840 (0.496 – 1.26) | <0.001 |
| 24 hours | 0.805 (0.426 – 1.46) | 0.001 |
| Treatment by time interaction |  | 0.677 |
| Treatment by time, per time point |  |  |
| GEL, 1 hour | 0.209 (-0.346 – 0.823) | 0.46 |
| GEL, 5 hours | 0.157 (-0.483 – 0.828) | 0.66 |
| GEL, 24 hours | 0.376 (-0.909 – 1.16) | 0.44 |
| Constant | 3.559 (3.06 – 4.13) | <0.001 |

**Neutrophil Gelatinase-Associated Lipocalin (adjusted for osmolality)**

Number of observations = 60

Number of subjects = 40

Observations per group = 4

| Predictor | β (95% confidence interval) | P value |
| --- | --- | --- |
| Treatment |  |  |
| CSL | Reference group |  |
| GEL | -0.246 (-0.968 – 0.476) | 0.51 |
| Time point |  |  |
| Baseline | Reference group |  |
| 1 hour | 0.262 (-0.186 – 0.710) | 0.25 |
| 5 hours | 0.574 (0.121 – 1.03) | 0.013 |
| 24 hours | 0.472 (-0.044 – 0.998) | 0.073 |
| Treatment by time interaction |  | 0.886 |
| Treatment by time, per time point |  |  |
| GEL, 1 hour | 0.180 (-0.308 – 0.668) | 0.47 |
| GEL, 5 hours | 0.115 (-0.447 – 0.680) | 0.69 |
| GEL, 24 hours | 0.213 (-0.534 – 0.961) | 0.58 |
| Osmolality | 0.002 (0.001 – 0.004) | 0.004 |
| Constant | 2.561 (1.767 – 3.355) | <0.001 |

**Cystatin C**

Number of observations = 60

Number of subjects = 40

Observations per group = 4

| Predictor | β (95% confidence interval) | P value |
| --- | --- | --- |
| Treatment |  |  |
| CSL | Reference group |  |
| GEL | -0.523 (-1.04 – 0.026) | 0.047 |
| Time point |  |  |
| Baseline | Reference group |  |
| 1 hour | 0.058 (-0.728 – 0.323) | 0.78 |
| 5 hours | 0.128 (-0.213 – 0.459) | 0.452 |
| 24 hours | 0.543 (0.109 – 0.92) | 0.009 |
| Treatment by time interaction |  | <0.001 |
| Treatment by time, per time point |  |  |
| GEL, 1 hour | 1.48 (1.08 – 2.28) | <0.001 |
| GEL, 5 hours | 2.29 (1.87 – 2.80) | <0.001 |
| GEL, 24 hours | 1.67 (0.961 – 2.36) | <0.001 |
| Constant | 2.97 (2.67 – 3.35) | <0.001 |

**Cystatin C (adjusted for osmolality)**

Number of observations = 60

Number of subjects = 40

Observations per group = 4

| Predictor | β (95% confidence interval) | P value |
| --- | --- | --- |
| Treatment |  |  |
| CSL | Reference group |  |
| GEL | -0.447 (-0.870 – -0.024) | 0.039 |
| Time point |  |  |
| Baseline | Reference group |  |
| 1 hour | -0.175 (-0.546 – 0.196) | 0.36 |
| 5 hours | -0.242 (-0.551 – 0.068) | 0.13 |
| 24 hours | 0.079 (-0.264 – 0.422) | 0.65 |
| Treatment by time interaction |  | <0.001 |
| Treatment by time, per time point |  |  |
| GEL, 1 hour | 1.44 (0.963 – 1.93) | <0.001 |
| GEL, 5 hours | 2.23 (1.82 – 2.64) | <0.001 |
| GEL, 24 hours | 1.44 (0.860 – 2.02) | <0.001 |
| Osmolality | 0.003 (0.002 – 0.004) | <0.001 |
| Constant | 1.58 (0.980 – 2.17) | <0.001 |
