# Supplementary figures and images for: A randomised controlled trial of succinylated gelatin (4%) fluid on urinary acute kidney injury biomarkers in cardiac surgical patients
Source: Intensive Care Med Exp. 2021 Sep 22;9:48. doi: 10.1186/s40635-021-00412-9 (PMC8455786; doi:10.1186/s40635-021-00412-9)

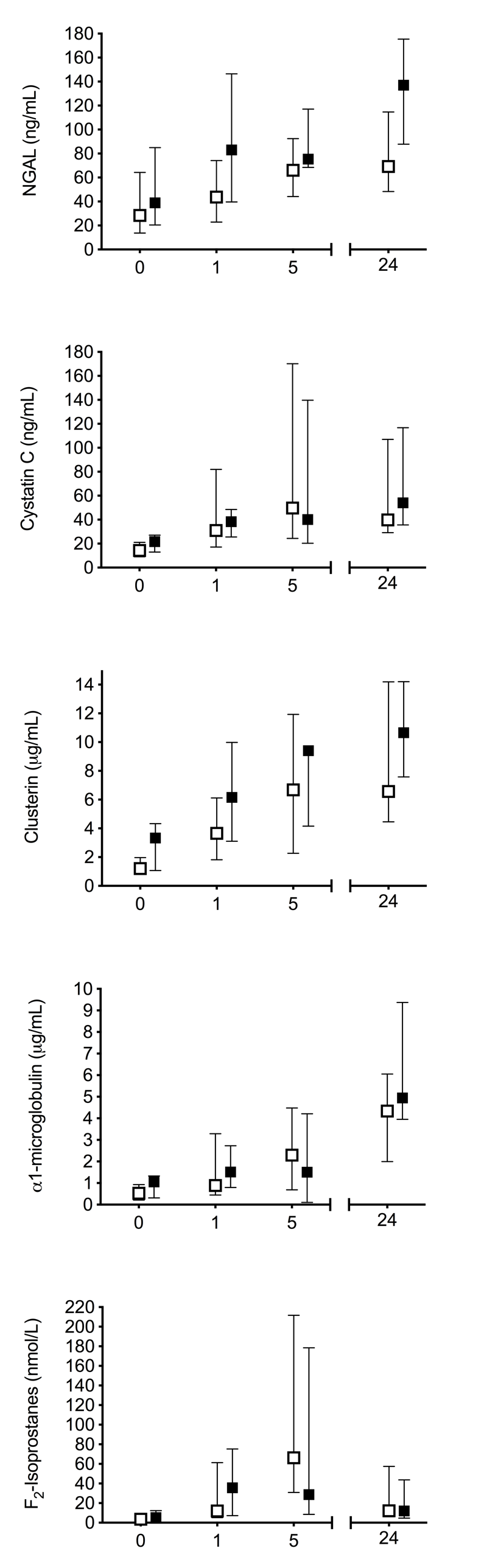

Supplement: Supplementary file 4 — Additional file 4. Urinary biomarker concentrations (median, Q1-Q3) NGAL, cystatin C, clusterin, α1-microglobulin and F2-isoprostanes of cardiac surgical patients who developed acute kidney injury (AKI) (closed squares) or did not develop AKI (open squares). AKI was defined as a maximum KDIGO score > 0 within 7 days of randomisation. Urine was sampled before the intervention (0 h), then 1, 5 and 24 h later. Abbreviations: AKI; acute kidney disease; KDIGO, Kidney Disease Outcomes Kidney Disease: Improving Global Outcomes; NGAL, neutrophil gelatinase-associated lipocalin. [file 40635_2021_412_MOESM4_ESM.tiff]
